# Acetaminophen Use: An Unusual Cause of Drug-Induced Pulmonary Eosinophilia

**DOI:** 10.1155/2016/4287270

**Published:** 2016-02-22

**Authors:** Mathieu D. Saint-Pierre, Onofre Moran-Mendoza

**Affiliations:** Division of Respirology and Critical Care Medicine, Department of Medicine, Queen's University, Kingston, ON, Canada K7L 2V6

## Abstract

Pulmonary eosinophilia (PE) can be found in very diverse pathological processes. Several medications have also been associated with this entity. Acetaminophen is a medication commonly used in multiple different drug formulations, many of which are available without a prescription. It has however been associated with pulmonary eosinophilia (eosinophilic pneumonia) in a few cases in Japan. We describe the case of a 68-year-old Caucasian female who presented with new persistent dry cough and dyspnea on exertion after she started using up to 4 grams of acetaminophen on a daily basis. Chest imaging revealed peripheral lower lung zone ground glass and reticular opacities, and increased eosinophils were present on bronchoalveolar lavage (BAL). The patient's symptoms markedly improved upon acetaminophen cessation, and significantly decreased eosinophils were seen on repeat BAL. To our knowledge, this is the first case of likely acetaminophen-induced pulmonary eosinophilia reported outside Japan.

## 1. Case Presentation

A 68-year-old Caucasian female presented with a 6-month history of new disabling nonproductive cough and exertional dyspnea (Medical Research Council dyspnea scale 2/5). She denied having any recent fevers, chills, or weight loss. The patient did not report new dermatologic complaints or arthralgias. She denied recent travel history. She had no history of smoking or drug use. She was retired, having previously worked in an office, and living in a well-kept home. Past medical history consisted of hypertension, idiopathic Raynaud's phenomenon, and mechanical back pain. Her medications included hydrochlorothiazide, which she had been on for seven years, vitamin D, calcium carbonate, and magnesium. Also, she was using everyday a drug formulation (Genacol® maximum strength) containing acetaminophen, chlorpheniramine, dextromethorphan, and pseudoephedrine for persistent nasal congestion, which she started just prior to the onset of her respiratory complaints. She had in addition began taking acetaminophen for a few months, for nonspecific “body aches,” up to a total combined amount of 4 grams per day. She had received two different courses of antibiotics from her family physician for her new respiratory symptoms as well as a trial of inhaled ciclesonide without any improvement, before being seen in Respirology Clinic.

On physical examination, the patient looked well. Her blood pressure was 140/70 mm Hg, heart rate 76 beats per minute, and SpO_2_ 97% on room air. Respiratory examination revealed the presence of fine bibasilar inspiratory crackles. She had no clubbing. The cardiovascular and abdominal examinations were unremarkable. A purple discoloration of the fingers and toes was present, in keeping with the patient's known Raynaud's phenomenon. The remainder of the examination was normal.

**Figure 1:**
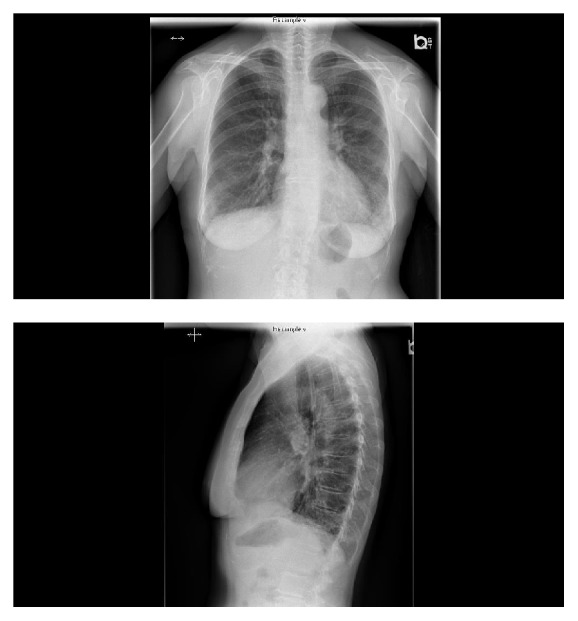
Posteroanterior and lateral chest X-ray revealing bilateral reticular infiltrates seen more significantly in the lower lung zones.

**Figure 2:**
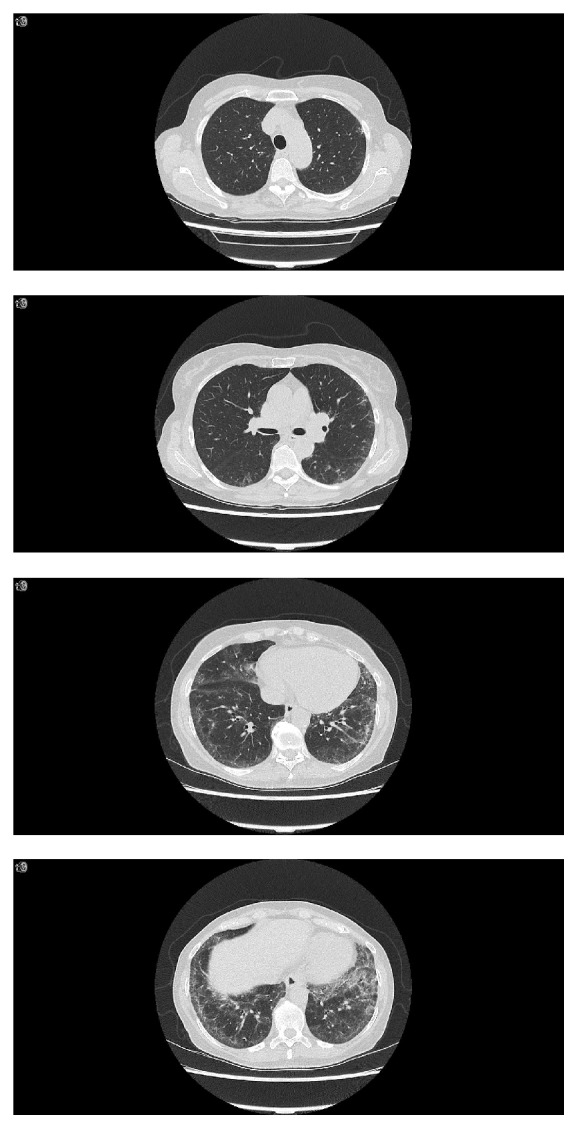
Chest computed tomography (CT) images showing lower lung zone predominant ground glass attenuation and reticulations present.

A chest radiograph showed increased bilateral lower lung interstitial markings (Figure 1). Chest computed tomography (CT) revealed bilateral peripheral ground glass opacities and reticulations involving the lower lung zones without honeycombing (Figure 2). On pulmonary function testing, her total lung capacity (TLC) was 4.71 L (94% of predicted), forced vital capacity (FVC) 2.55 L (91% of predicted), forced expiratory volume in one second (FEV1) 2.37 L (118% of predicted), and her FEV1/FVC ratio 93%. There was no significant FEV1 change after bronchodilator. Diffusion capacity of carbon monoxide (DLCO) was normal at 76% of predicted.

The complete blood cell count was normal: hemoglobin 141 gr/L, white blood cell count 8.1 × 10^9^/L, eosinophils 0.1 × 10^9^/L, and platelet count 232 × 10^9^/L. Electrolytes, urinalysis, and renal and liver function tests were normal. Erythrocyte sedimentation rate and C-reactive protein were mildly elevated, at 28 mm/H and 5.4 mg/L, respectively. Antinuclear antibody level was weakly positive at 1 : 40, with a speckled pattern; the extractable nuclear antigens panel was negative. Complement levels, anti-double-stranded DNA, rheumatoid factor, anti-cyclic citrullinated protein antibody, cryoglobulins, perinuclear anti-neutrophil cytoplasmic antibodies (ANCA), and cytoplasmic ANCA were all either normal or negative. The patient was assessed by rheumatology; no evidence of a connective tissue disease was found.

Hydrochlorothiazide was stopped due to reported cases of drug-induced pneumonitis [1], and the patient was started on amlodipine. The patient's respiratory complaints had not improved when seen in follow-up and repeat chest CT showed interval worsening of the bilateral infiltrates. Bronchoscopy was thus performed to better define the diagnosis. Bacterial, mycobacterial, and fungal cultures were negative. Cytology was unrevealing. BAL cell count differential showed significantly elevated eosinophils at 29%. Transbronchial biopsies displayed only mild nonspecific fibrosis.

The patient was seen again in clinic after the bronchoscopy. A new literature search was performed and revealed a rare but previously described association between acetaminophen use and pulmonary eosinophilia [1]. None of her other medications had been associated with PE. The patient was still taking up to 4 grams of acetaminophen per day and was told to stop doing so. Marked improvement of her cough and complete resolution of the patient's dyspnea were seen within a few days. The residual cough was very mild, occurring upon waking up, and attributed to postnasal drip by the patient when she was assessed in clinic three months later. The patient subsequently agreed to have a repeat bronchoscopy performed; BAL eosinophils had decreased to 12%.

## 2. Discussion

Pulmonary eosinophilia, also called eosinophilic pneumonia or pulmonary infiltrates with eosinophilia, can be caused by drugs and toxins; it has also been described in autoimmune, infectious, and malignant processes or can be idiopathic [2]. The list of medications reported to cause PE is extensive. Nonsteroidal anti-inflammatory drugs and antimicrobials are the most common drugs, but a number of other agents have been implicated [1]. Drugs also can produce “Drug Rash with Eosinophilia and Systemic Symptoms” that includes internal organ involvement in addition to skin rash and peripheral eosinophilia; respiratory involvement occurs in about 15% of patients [1]. Ingestion of L-tryptophan and inhalation of smoke, heroin, crack cocaine, and marijuana have been associated with PE [1], as well as conditions such as eosinophilic granulomatosis with polyangiitis (Churg-Strauss syndrome), allergic bronchopulmonary aspergillosis, systemic hypereosinophilic syndromes, and Langerhans cell histiocytosis. It has been described with bacterial, viral, parasitic, and fungal infections. When no cause is found, the entity has been called idiopathic eosinophilic pneumonia, either acute or chronic. Asthma, eosinophilic bronchitis, idiopathic pulmonary fibrosis, sarcoidosis, hypersensitivity pneumonitis, cryptogenic organizing pneumonia, and connective tissue diseases can be associated with modest degrees of eosinophilia on BAL (<25%) [2].

Pulmonary eosinophilia can present with nonproductive cough, dyspnea, hypoxemia, fever, malaise, and weight loss. Peripheral blood eosinophilia is often seen but may be absent. Pulmonary function tests frequently show a restrictive pattern; concomitant airway obstruction is seen in some cases. CT findings of drug-induced PE almost always include areas of ground glass attenuation and airspace consolidation, with a lower lung or random zonal predominance. Also, pulmonary infiltrates tend to be more peripheral than central in distribution [3]. Clinical presentation, physical examination, bloodwork, and chest imaging may permit distinguishing PE from other clinical entities. If diagnostic uncertainty remains after noninvasive investigations, bronchoscopy should be considered. A BAL with eosinophils ≥25% in the cell count differential usually confirms the diagnosis of pulmonary eosinophilia [2]. Identifying an offending drug or toxin is of significant relevance as respiratory complaints often subside after its discontinuation without any other therapy; in severe or refractory cases, systemic corticosteroids should be considered.

Acetaminophen is a very common medication used for its analgesic and antipyretic properties. It is also found in many formulations often available over the counter, which may contain antihistamines or cough suppressants. Patients are often prone to forgetting to mention acetaminophen use when seeing their physician given it is widely available. It has however been linked with the development of eosinophilic lung disease in five cases from Japan [4–8], the first published in 1992. Patients usually reported a fairly acute onset of symptoms shortly after acetaminophen exposure with the development of reticulonodular opacities on chest X-rays and ground glass opacities in the only case that had chest CT done [8]. All patients improved after drug cessation [4–8]. In our case, although medication rechallenge would have been required to confidently confirm the diagnosis, we believe that the presentation was caused by high-dose acetaminophen: the patient's symptoms started shortly after initiation, the chest CT showed reticulation and ground glass opacities with lower lobe predominance, the BAL demonstrated a high eosinophil count, and her symptoms and pulmonary eosinophilia markedly improved after drug discontinuation.

To our knowledge, this is the first case of likely acetaminophen-induced pulmonary eosinophilia reported outside Japan and is a reminder to consider common medications as a cause of drug-induced PE.

## Learning Objectives

Learning objectives are as follows:

- To review the diagnosis and main conditions associated with pulmonary eosinophilia (PE).
- To recognize that common medications, including acetaminophen, have been linked with pulmonary eosinophilia.

*CanMEDS Competency.* The CanMEDS role is Medical Expert.

*Pretest.* It includes the following.

- What are the main conditions associated with pulmonary eosinophilia?
- How is drug-induced pulmonary eosinophilia diagnosed and treated?

*Posttest.* It includes the following.

*(i) What Are the Main Conditions Associated with Pulmonary Eosinophilia?* Pulmonary eosinophilia can be caused by drugs and toxins; it has also been described in autoimmune, infectious, and malignant processes or can be idiopathic.

*(ii) How Is Drug-Induced Pulmonary Eosinophilia Diagnosed and Treated?* Drug-induced PE can present with nonproductive cough and/or dyspnea, hypoxemia, and infectious or constitutional symptoms. Chest CT findings of drug-induced PE include ground glass attenuation and airspace consolidation, with a lower lung or random zonal predominance. The presence of ≥25% eosinophils on bronchoalveolar lavage confirms the diagnosis of pulmonary eosinophilia. The main treatment involves discontinuation of the drug; in severe cases or when a patient does not improve after discontinuation of the drug, systemic steroids should be considered.

*Additional Reference.* See [9].
